# Using CADD tools to inhibit the overexpressed genes FAP, FN1, and MMP1 by repurposing ginsenoside C and Rg1 as a treatment for oral cancer

**DOI:** 10.3389/fmolb.2023.1248885

**Published:** 2023-10-23

**Authors:** Manal Abouelwafa, Tamer M. Ibrahim, Mohamed S. El-Hadidi, Mater H. Mahnashi, Amani Y. Owaidah, Nizar H. Saeedi, Hany G. Attia, John J. Georrge, Amany Mostafa

**Affiliations:** ^1^ Department of Bioinformatics, Christ College, Rajkot, Gujarat, India; ^2^ Department of Pharmaceutical Chemistry, Faculty of Pharmacy, Kafrelsheikh University, Kafrelsheikh, Egypt; ^3^ Bioinformatics Group, Center for Informatics Sciences, School of Information Technology and Computer Science, Nile University, Giza, Egypt; ^4^ Department of Pharmaceutical Chemistry, College of Pharmacy, Najran University, Najran, Saudi Arabia; ^5^ Department of Clinical Laboratory Sciences, College of Applied Medical Sciences, Imam Abdulrahman bin Faisal University, Dammam, Saudi Arabia; ^6^ Department of Medical Laboratory Technology, Faculty of Applied Medical Sciences, University of Tabuk, Tabuk, Saudi Arabia; ^7^ Department of Pharmacognosy, College of Pharmacy, Najran University, Najran, Saudi Arabia; ^8^ Department of Bioinformatics, University of North Bengal, West Bengal, India; ^9^ Nanomedicine and Tissue Engineering Laboratory, Medical Research Centre of Excellence, National Research Centre (NRC), Cairo, Egypt

**Keywords:** FAP, FN1, MMP1, ginsenoside C and Rg1, molecular docking, simulation

## Abstract

Oral cancer is one of the most common cancer types. Many factors can express certain genes that cause the proliferation of oral tissues. Overexpressed genes were detected in oral cancer patients; three were highly impacted. FAP, FN1, and MMP1 were the targeted genes that showed inhibition results *in silico* by ginsenoside C and Rg1. Approved drugs were retrieved from the DrugBank database. The docking scores show an excellent interaction between the ligands and the targeted macromolecules. Further molecular dynamics simulations showed the binding stability of the proposed natural products. This work recommends repurposing ginsenoside C and Rg1 as potential binders for the selected targets and endorses future experimental validation for the treatment of oral cancer.

## 1 Introduction

Computer-aided drug design (CADD) is a more time-efficient and cost-effective method than traditional drug-design techniques. In the past, it could take approximately 13 years to develop a treatment (Wooller, Benstead-Hume, Chen, Ali and Pearl, 2017). Drug targets could be enzymes, proteins, or genes. These targets must either be inhibited in the case of over-expression or activated in the case of downregulation (Smith et al., 2010; Rufer, 2021). Finding ligands for particular targets could help in the next stage of drug discovery *in vivo* and in clinical trials (Togre, Vargas, Bhargavi, Mallakuntla and Tiwari, 2022). Recently, CAAD has become more developed and involves artificial intelligence and machine learning (Deng, Yang, Ojima, Samaras and Wang, 2022).

For centuries, drug discovery was a very long and complex method that cost substantial money and time, and led the loss of patients during experiments. After discovering *in silico* studies, the impact on humanity was significant. Due to the increasing number of diseases, researchers could develop drug discovery methods. Molecular docking is one of the most effective drug discovery tools. It is a way to find out the mechanism of the ligand or molecule in defense of the disease. Many CAAD tools are available to predict adverse effects, target fishing and profiling, drug repurposing, and the prospective drug target (Pinzi and Rastelli, 2019).

Small molecules are docked into macromolecular structures using a method known as molecular docking to score their complementary values at the binding sites (Saikia and Bordoloi, 2019). Many CAAD tools can accurately model and predict target protein’s active sites. Molecular docking strategies, such as structure and ligand-based methods, are used in drug discovery (Ferreira, Dos Santos, Oliva and Andricopulo, 2015). More attractive and well-designed tools will be available as long as there is a demand for drug discovery. Since it provides a more significant percentage of the interaction, the simulation approach was created as a technique for drug design. The physiological environment can demonstrate the true stability of the binding sites for ligands and macromolecules (Lin et al., 2020).

Precancer describes the signs in a normal cell that could transform to cancer. (Lopez-Lopez et al., 2015). searched pre-oral cancer lesion prevention keywords from 2005 to 2015 and clinical trials from January 2011 to 2015. The authors referred to the less significant patterns between pre-oral cancer lesions and early diagnosis (Lopez-Lopez, Omana-Cepeda and Jane-Salas, 2015). However, some oral conditions might present before oral cancer, such as erythroplakia, leukoplakia, lichen planus, and submucosal fibrosis (Abati, Bramati, Bondi, Lissoni and Trimarchi, 2020). The normal oral microbial flora is pivotal in maintaining a normal oral physiological environment. On the other hand, insufficient self-hygiene can lead to oral diseases like periodontal and tooth loss. In a small number of cases, the oral microbiome can be developed into oral cancer or a chronic disease. The oral cavity can contain over 700 microbial species such as bacterium, fungi, and viruses, which can change the mechanism of cells by unhealthy practices (chewing tobacco) and cause oral cancer, similar to the case of *H. Pylori* causing gastric cancer (Gholizadeh et al., 2016). *Candida* is one of these fungi infections, which is very normal, but in abnormal circumstances, it can cause oral cancer (Tarapan et al., 2019). Another type of risk factor for oral cancer is herpes simplex virus (HSV), which can form oral squamous cell carcinomas and interfere with the cell cycle by encoding viral oncoproteins. The mechanism of this development is that HSV1 causes ocular and oral infections, and HSV2 leads to genital infections (D'Souza and Addepalli, 2018). All these risk factors can lead to oral cancer, but one cannot be sure that these factors are the main reason for oral cancer.

The frequency of oral cancer has increased over the past 10 years, and it is typically diagnosed later when symptoms first appear. According to data from the Global Cancer Observatory (GCO), there were 377,713 new cases of oral squamous cell carcinoma (OSCC) reported annually in the world in 2020, with Asia recording the highest number of cases (248,360), followed by Europe (65,279) and North America (27,469) (Deng et al., 2022). Cases found in young patients were linked to alcohol and tobacco use, and insufficient self-care habits (Macfarlane et al., 1994; Sciubba, 2001; Ali et al., 2017; Ghantous and Elnaaj, 2017). The estimated 5-year overall survival rate for oral cancer has stayed at a dismal 50% over the past several decades. It has been among the worst cancer death rates, being much lower than cancer of colorectal, cervical, and breast origin. The lack of early detection and diagnosis mainly causes this. Although there have been considerable advancements in cancer treatment, the best method to preserve patients’ lives and enhance their quality of life is through early detection of oral cancer and its treatable antecedents (Napier and Speight, 2008; van der Waal, 2010). FAP, FN1, and MMP1 are overexpressed as well as playing a vital role in anti-apoptosis and leading to oral cancer (Nariai, Mishima, Yoshimura and Sekine, 2011; Ying et al., 2022). Moreover, FN1 could affect the immune response in oral cancer cells (Peng et al., 2023).

This study aimed to learn more about oral cancer and discover a cutting-edge treatment. One of the most crucial techniques in drug design is repurposing already approved medications. We currently have three possible therapeutic targets that may influence future research on oral cancer. One of the most used techniques in the pharmaceutical industry is computer-aided drug discovery. The study provides a novel therapy strategy for oral cancer, for which, considered to be one of the most severe diseases, there is little knowledge of effective treatments. More research is necessary to stop the rising incidence of oral cancer patients.

## 2 Materials and methods

### 2.1 Retrieval of protein 3D structure

Many genes can affect oral cancer expression. These genes can be up-regulated or down-regulated. When overexpressed, they enhance the cancer cells. Fibroblast activation protein (FAP), Fibronectin 1 (FN1), and Matrix metalloproteinase-1 (MMP1) are overexpressed genes that are associated with oral cancer prognosis (Syed et al., 2020; Zhang et al., 2022; Zhou et al., 2022). The FAP, FN1, and MMP1 proteins were obtained from the Protein Data Bank (PDB) (https://www.rcsb.org/), one of the largest databases for 3D structures (Berman et al., 2000). FAP, FN1, and MMP1 proteins were identified as PDB IDs: 1z68, 3m7p, and 3shi, respectively.

### 2.2 Ligand construction

A total of 2021 approved drugs were retrieved from the DrugBank (https://go.drugbank.com/) database. The approved drugs can be repurposed, which means one drug can be used for more than one disease. This drug database is a bioinformatics/cheminformatics resource that combines drug data with comprehensive drug target information (Wishart et al., 2006). LigPrep modules of Schrodinger version 12.8 prepared the ligands. The OPL4 force field was used to prepare the ligands.

### 2.3 Molecular docking

Molecular docking studies were performed using the virtual screening workflow of Schrodinger version 12.8. The top 10% of molecules from each step, high-throughput virtual screening (HTVS), standard precision (SP), and extra precision (XP), were screened out, and the results were analyzed.

### 2.4 Molecular dynamics

Molecular dynamics simulation was done using Desmond v. 12 to examine the stability of the interaction of the molecules in the complexes FAP protein and ginsenoside C (1z68- DB06748), FAP protein and Rg1 (1z68-DB06750), FN1 protein and ginsenoside C (3m7p-DB06748), and MMP1 protein and ginsenoside C (3shi-DB06748).

One of the quickest simulation tools is Desmond of Schrödinger, which runs at a rate of 471 nanoseconds per day on the 1,024 cores of the InfiniBand cluster (Chow et al., 2008). Designing proteins with capped termini and hydrogen bonds required Maestro 12.7. Desmond was used to do simulations and molecular minimization using default settings (Li et al., 2019). The system designed to limit the shape of water molecules and heavy atom bond lengths with hydrogen used the counter ions shake technique. Using the Particle Mesh Ewald (PME) approach and orthorhombic equations, the electrostatic interaction was implemented as periodic boundary conditions (PBC) (Srivastava, Garg, Srivastava and Seth, 2021). It was important to choose the protein-ligand with the lowest binding energy.

## 3 Results

### 3.1 Molecular docking

**FIGURE 1:**
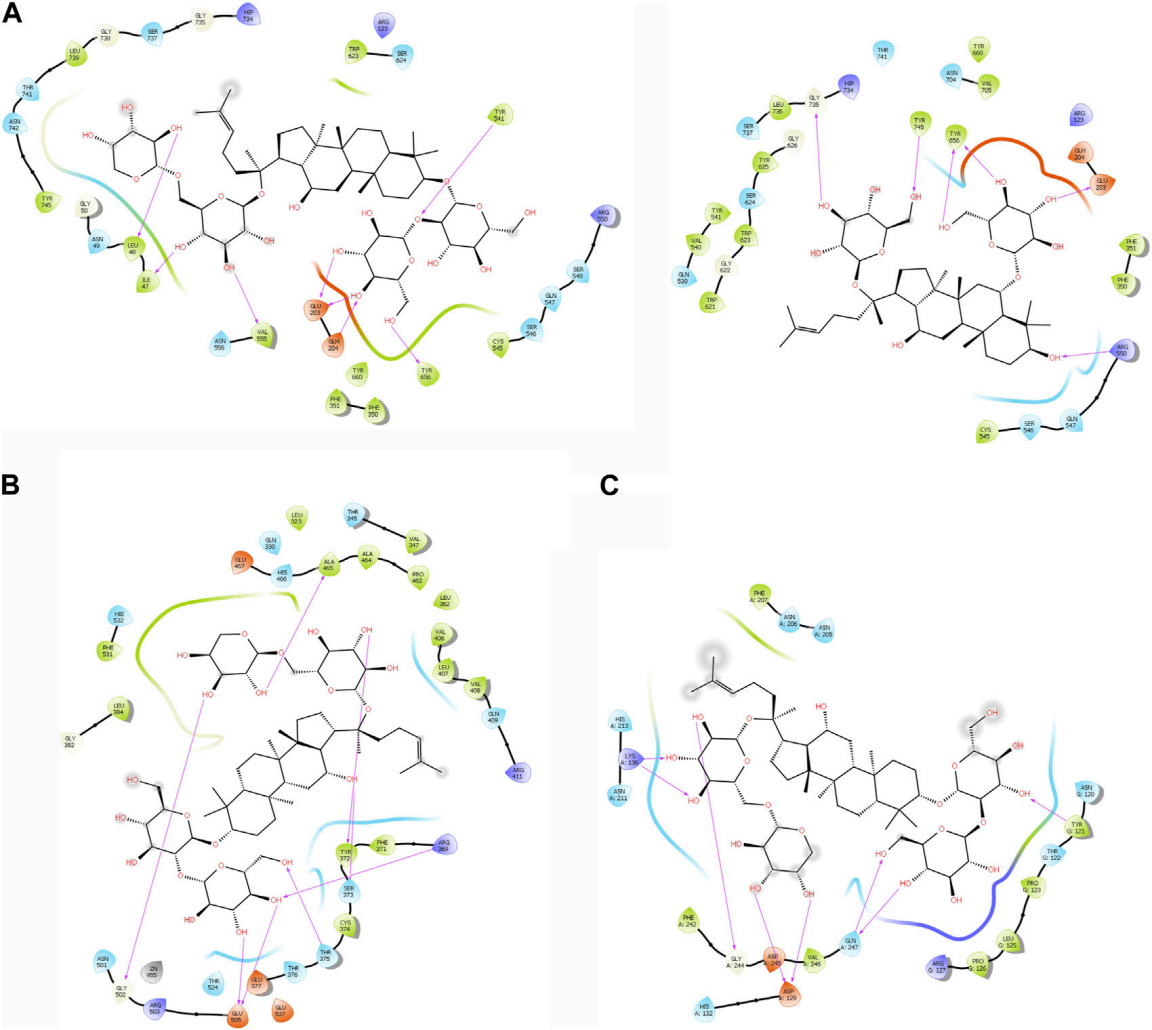
**(A)**: The interaction with FAP protein, ginsenoside C, and Rg1 showing the amino acids **(B)**: The interaction between FN1 protein and ginsenoside C showing the amino acids **(C)**: The interaction between MMP1 protein and ginsenoside C showing the amino acids.

The purpose of including this database is to repurpose approved drugs to cure oral cancer. After complete docking with all approved drugs, the results were very promising. They showed that ginsenoside molecules interacted well with the three proteins with good docking scores. In addition, ginsenoside C (DB06748) interacted with all proteins as FAP (−12.142 kcal/mol), FN1 (−14.142 kcal/mol), and MMP1 (−9.415 kcal/mol). Moreover, DB06750 (ginsenoside Rg1) interacted with FAP with the best score (−11.303 kcal/mol). Supplementary Table S1; Figure 1A show the amino acid interaction FAP protein with ginsenoside C and Rg1 (1z68- DB06748 and 1z68-DB06750) **1 (B)** show the amino acid interaction between FN1 protein and ginsenoside C (3m7p-DB06748)**, and 1 (C)** show the amino acid interaction between MMP1 protein and ginsenoside C (3shi-DB06748).

### 3.2 Molecular dynamics

#### 3.2.1 Protein information

**FIGURE 2:**
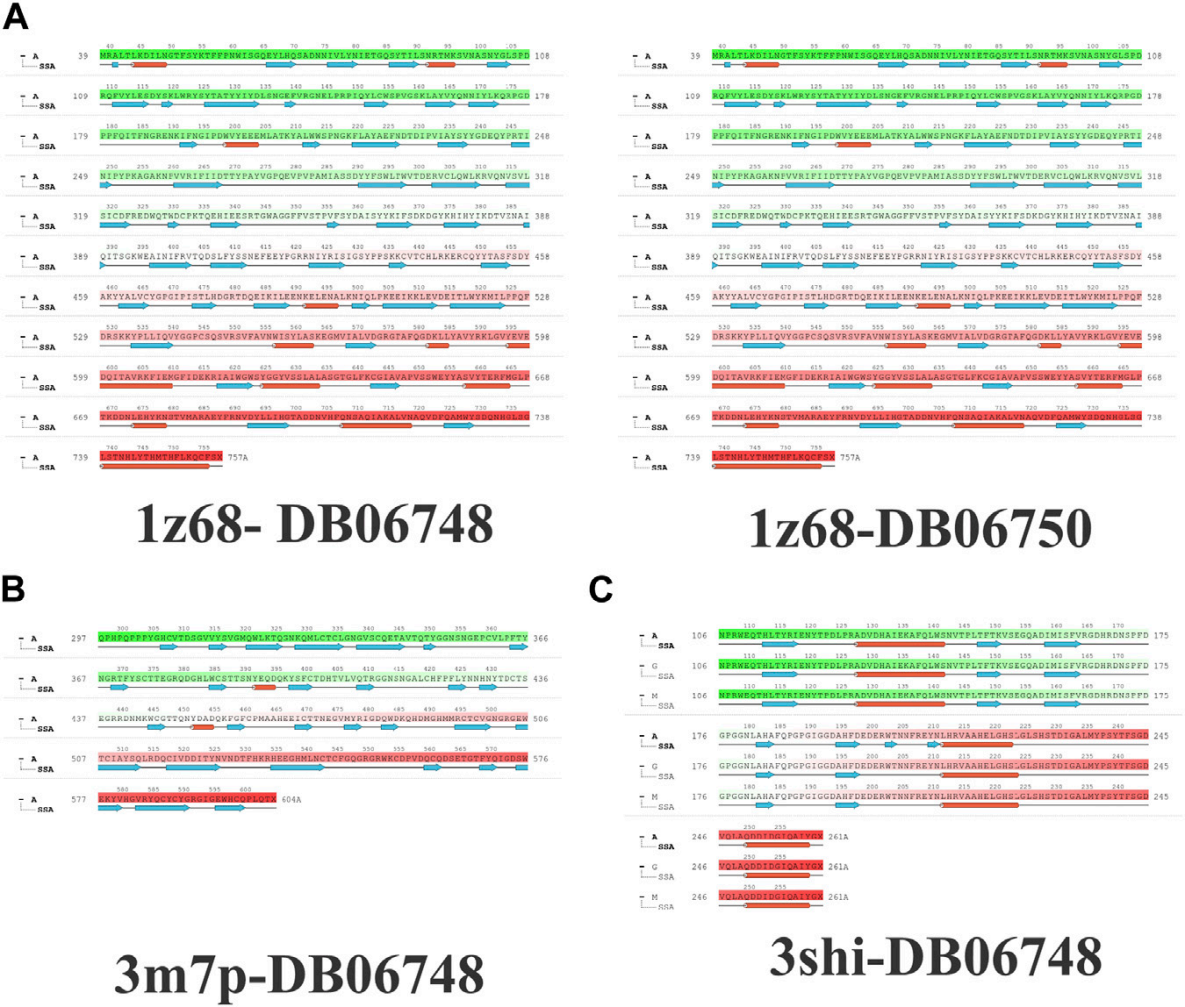
**(A–C)**: protein total residues, chain atoms, heavy atoms, and charged atoms for the four complexes.

**FIGURE 3:**
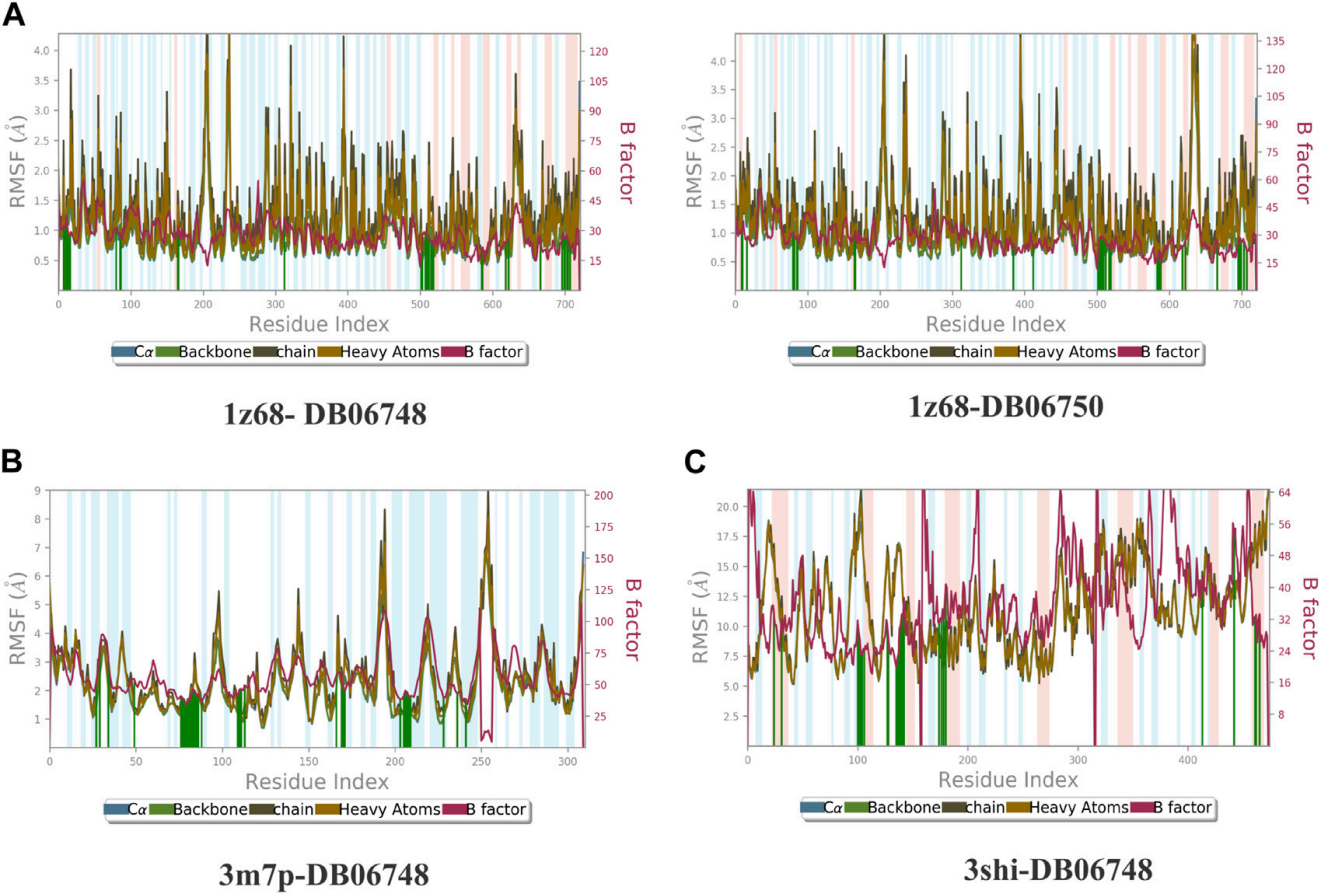
**(A–C)**: Root mean square fluctuation (RMSF) for four complexes in which the mean is not over 2.5 A°.

**FIGURE 4:**
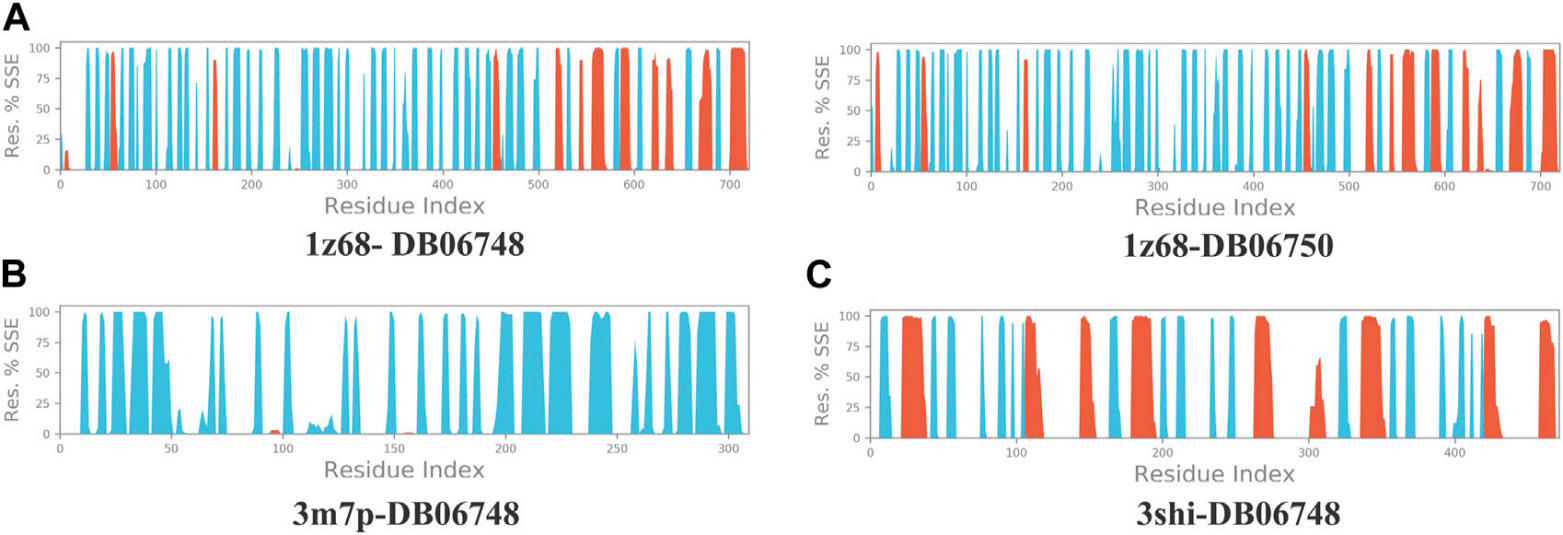
**(A–C)**: Protein secondary structure elements (SSE); alpha shown in red and beta strands shown in blue.

Figures 2A–C depicts the total protein residues, and chain, heavy, and charged atoms for the four complexes 1z68-DB06748, 1z68-DB06750, 3m7p-DB06748, and 3shi-DB06748, respectively. The RMSF A° (root mean square fluctuation) for the three targeted proteins (FAP, FN1, and MMP1) with (Ginsenoside C and Rg1) ligands is not greater than 2.5 A°, as shown in Figures 3A–C. Furthermore, Figures 4A–C depicts the protein’s secondary structure elements, with alpha strands in red and beta strands in blue.

#### 3.2.2 Ligand information

**FIGURE 5:**
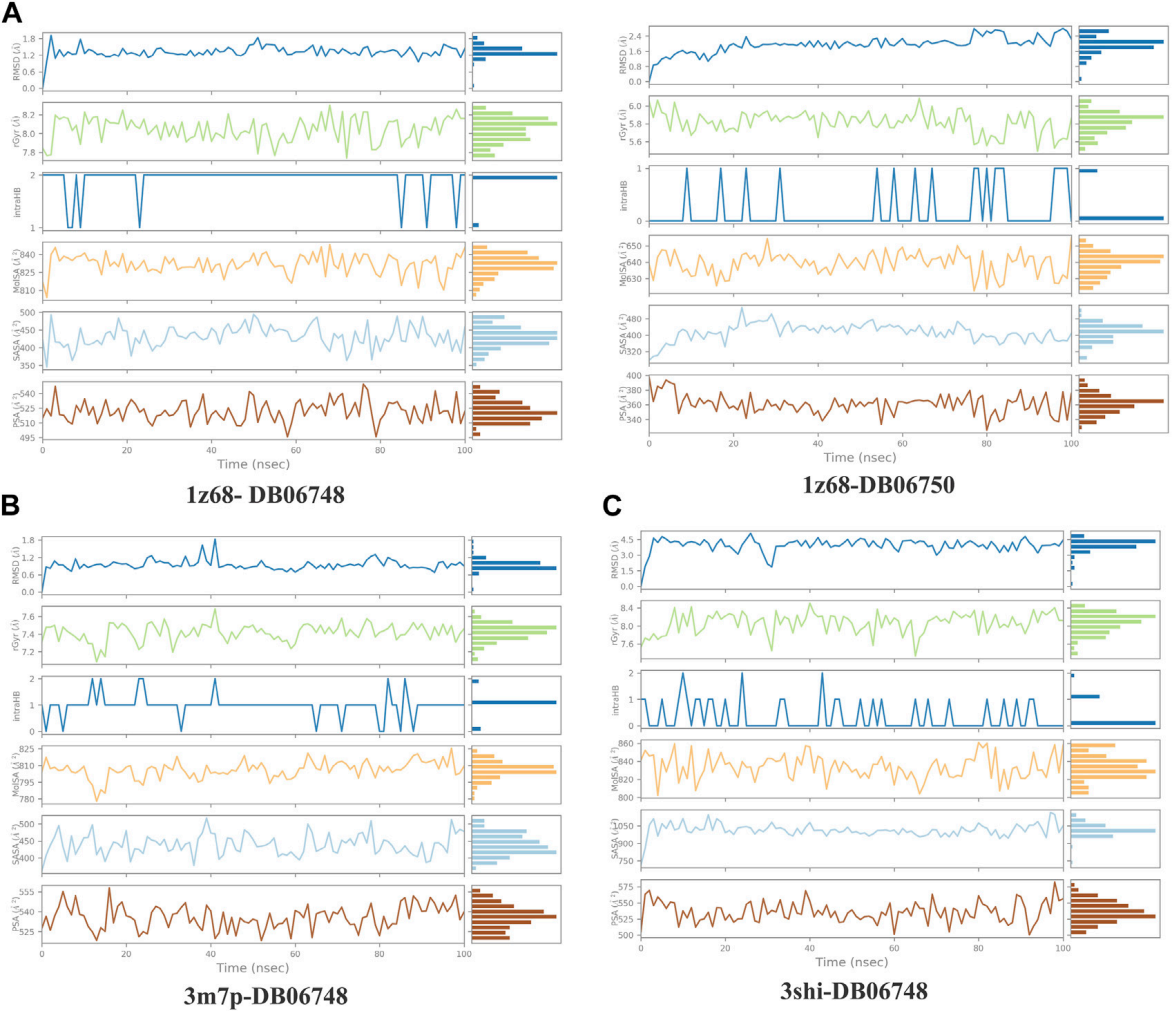
**(A–C)**: ligand properties for four ligands as root mean square (RMSD) for ligands, radius of gyration (rGYr), intramolecular hydrogen bonds (intra HB), molecular surface area (MoISA), solvent accessible surface area (SASA), and polar surface area (PSA).

**FIGURE 6:**
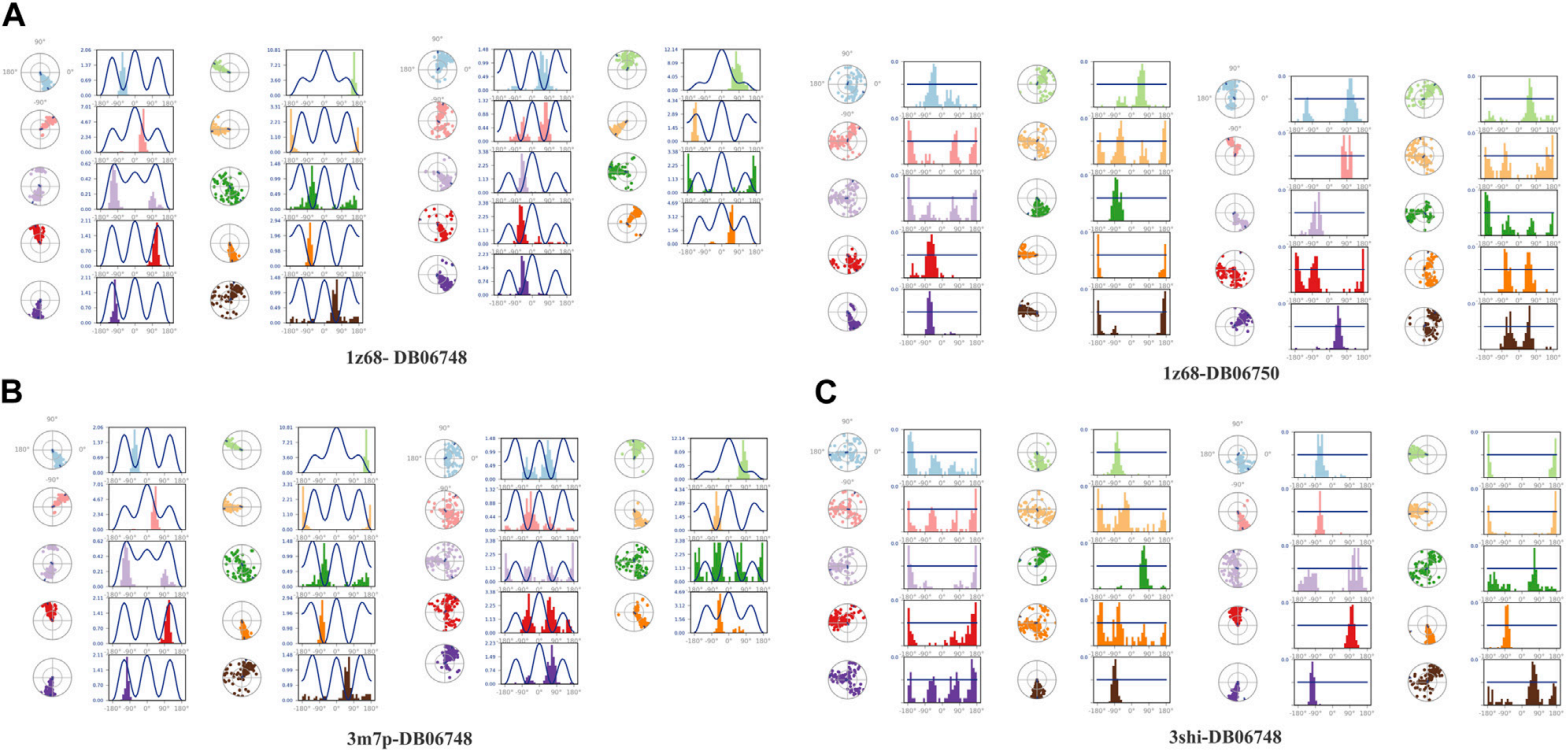
**(A–C)**: Ligand’s torsion plot.

Figures 5A–C displays the properties of four ligands, including the root mean square (RMSD) for the ligands, the radius of gyration (rGYr), the number of intramolecular hydrogen bonds (intra HB), the molecular surface area (MoISA), the solvent accessible surface area (SASA), the surface area of a molecule that is accessible by a water molecule, and the polar surface area (PSA). The ligand torsion diagram shown in Figures 6A–C depicts the conformational evolution of each rotatable bond (RB) along the simulation trajectory (0.00–100 nsec).

**FIGURE 7:**
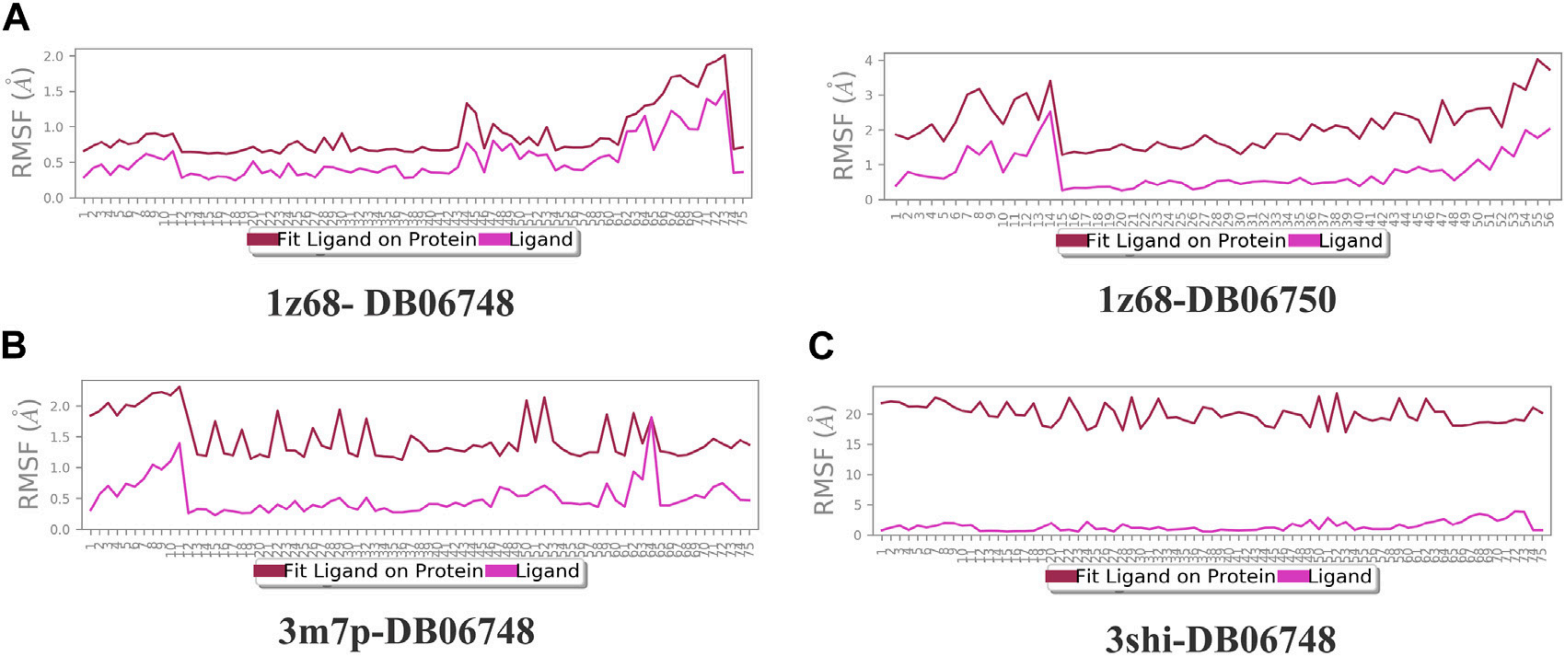
**(A–C)**: The ligand root mean square fluctuation (L-RMSF) for the four complexes.

Figures 7A–C illustrates the calculation of root mean square (L-RMSF) to maintain the changes in the locations of the ligand atoms. According to the 2D structure in the top panel, L-RMSF displays the ligand’s fluctuations broken down by the atom. We can understand how ligand fragments interact with proteins and have an entropic role in the binding event by using the ligand RMSF. The “Fit Ligand on Protein” line in the bottom panel displays the ligand fluctuations concerning the protein. The protein-ligand complex is first aligned on the protein backbone before measuring the ligand RMSF on the ligand-heavy atoms. The “Ligand” line depicts fluctuations where the ligand in each frame is lined up with the ligand in the reference frame. The fluctuations of this line are measured for the ligand-heavy atoms. These RMSF values represent the ligand’s internal atom variations.

**FIGURE 8:**
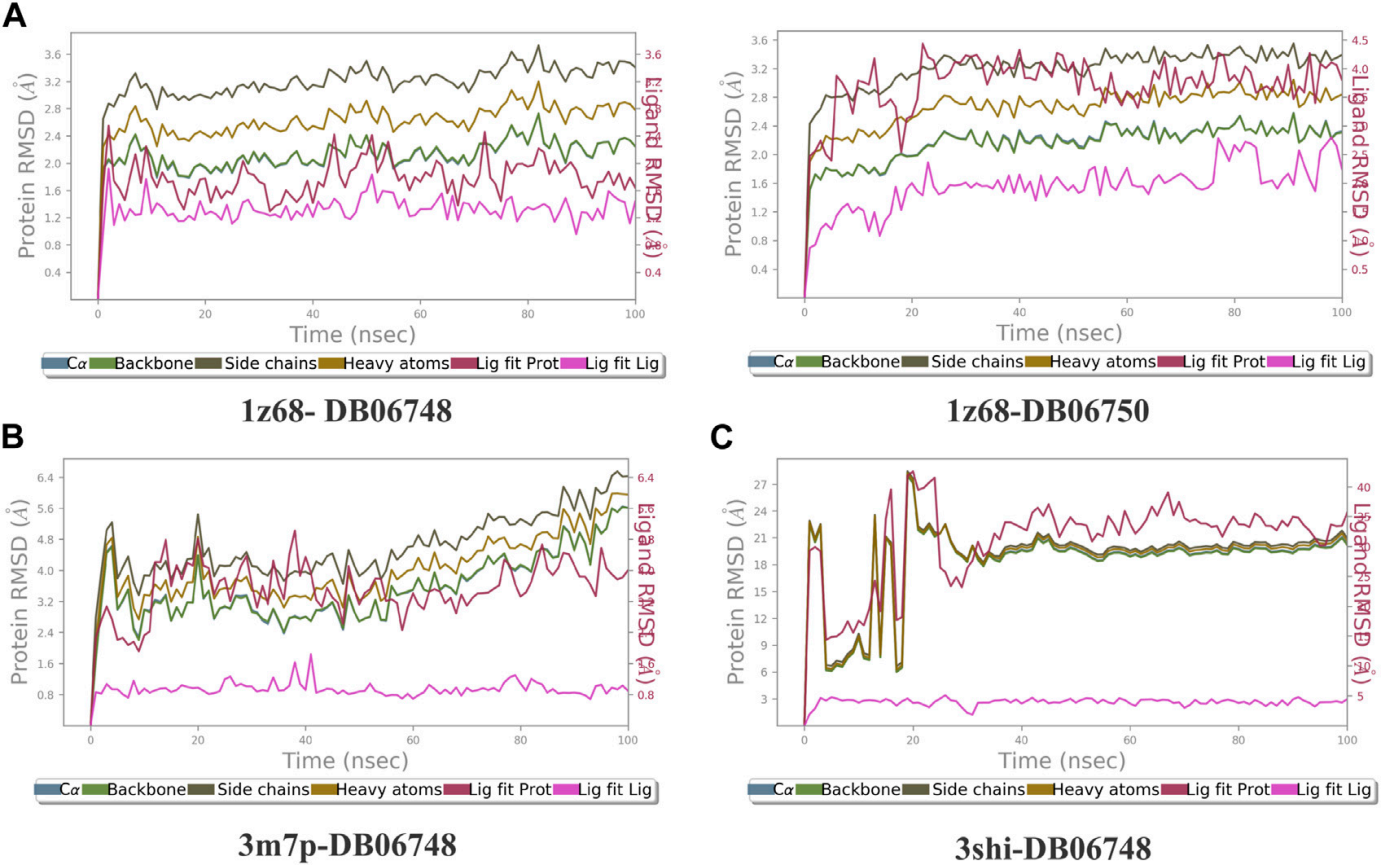
**(A–C)**: The Root Mean Square Deviation (RMSD) for the four complexes Protein RMSD: The above plot shows the RMSD evolution of a protein (left Y-axis). All protein frames are first aligned on the reference frame backbone, and then the RMSD is calculated based on the atom selection. Ligand RMSD: Ligand RMSD (right Y-axis) indicates how stable the ligand is regarding the protein and its binding pocket. In the above plot, ‘Lig fit Prot’ shows the RMSD of a ligand when the protein-ligand complex is first aligned on the protein backbone of the reference, and then the RMSD of the ligand heavy atoms is measured.

**FIGURE 9:**
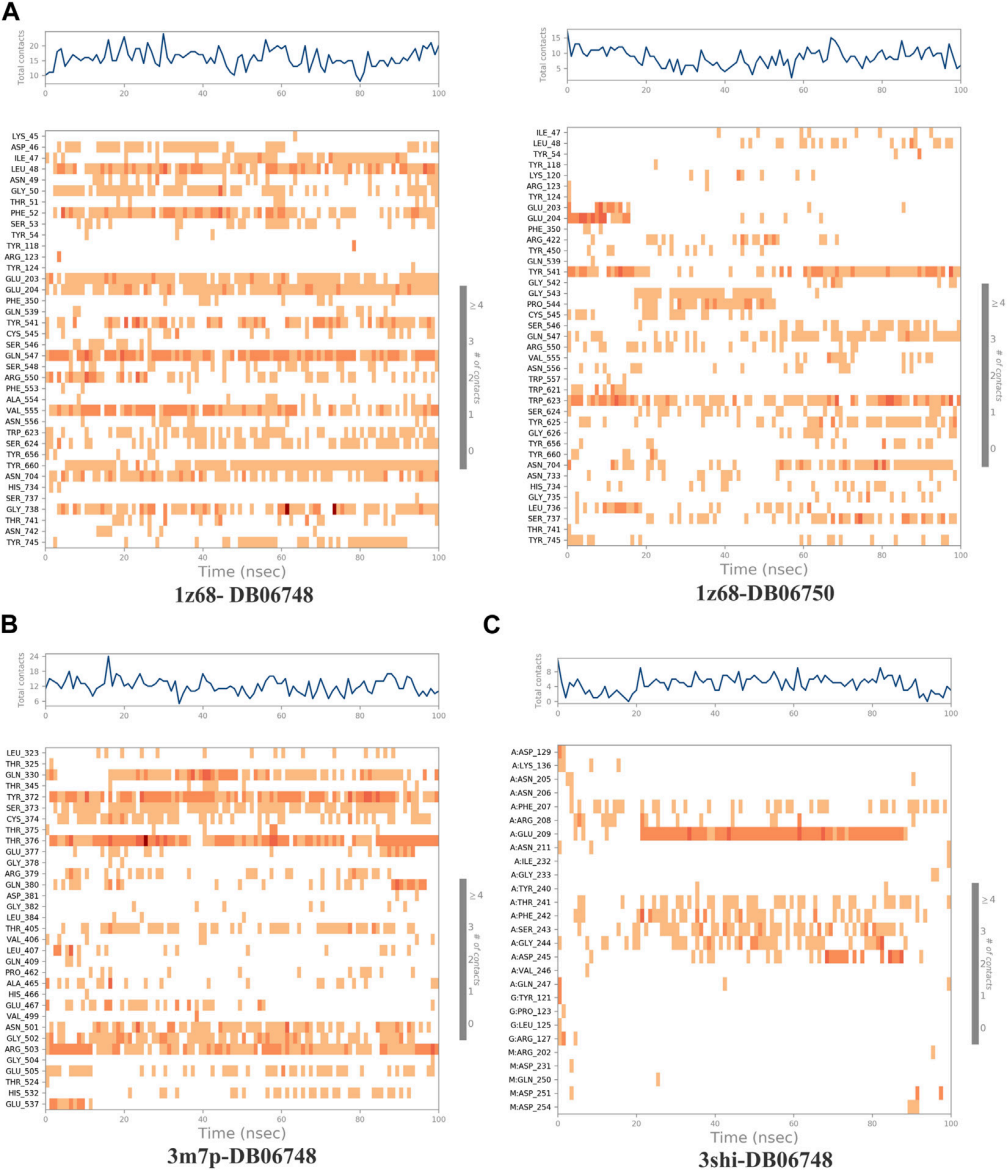
**(A–C)**: A timeline representation of the interactions and contacts (H-bonds, Hydrophobic, Ionic, Water bridges) of all four complexes.

**FIGURE 10:**
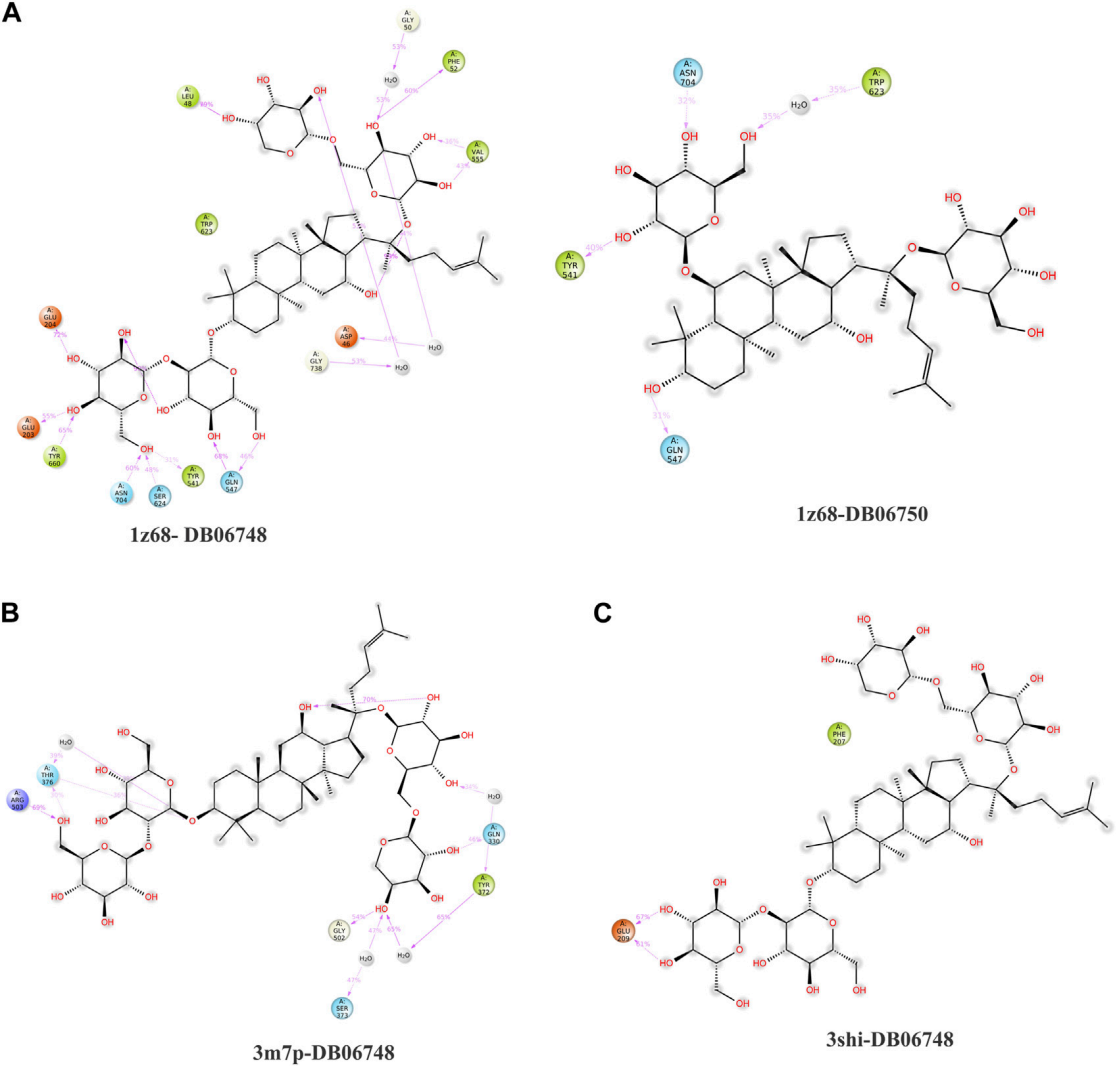
**(A–C)**: Protein-ligand interactions (or ‘contacts’) are categorized into Hydrogen Bonds, Hydrophobic, Ionic and Water Bridges. Each interaction type contains more specific subtypes, which can be explored through the ‘Simulation Interactions Diagram’ panel. The stacked bar charts are normalized throughout the trajectory: for example, a value of 0.7 suggests that the specific interaction is maintained for 70% of the simulation time. Values over 1.0 are possible as some protein residue may make multiple contacts of the same subtype with the ligand.

**FIGURE 11:**
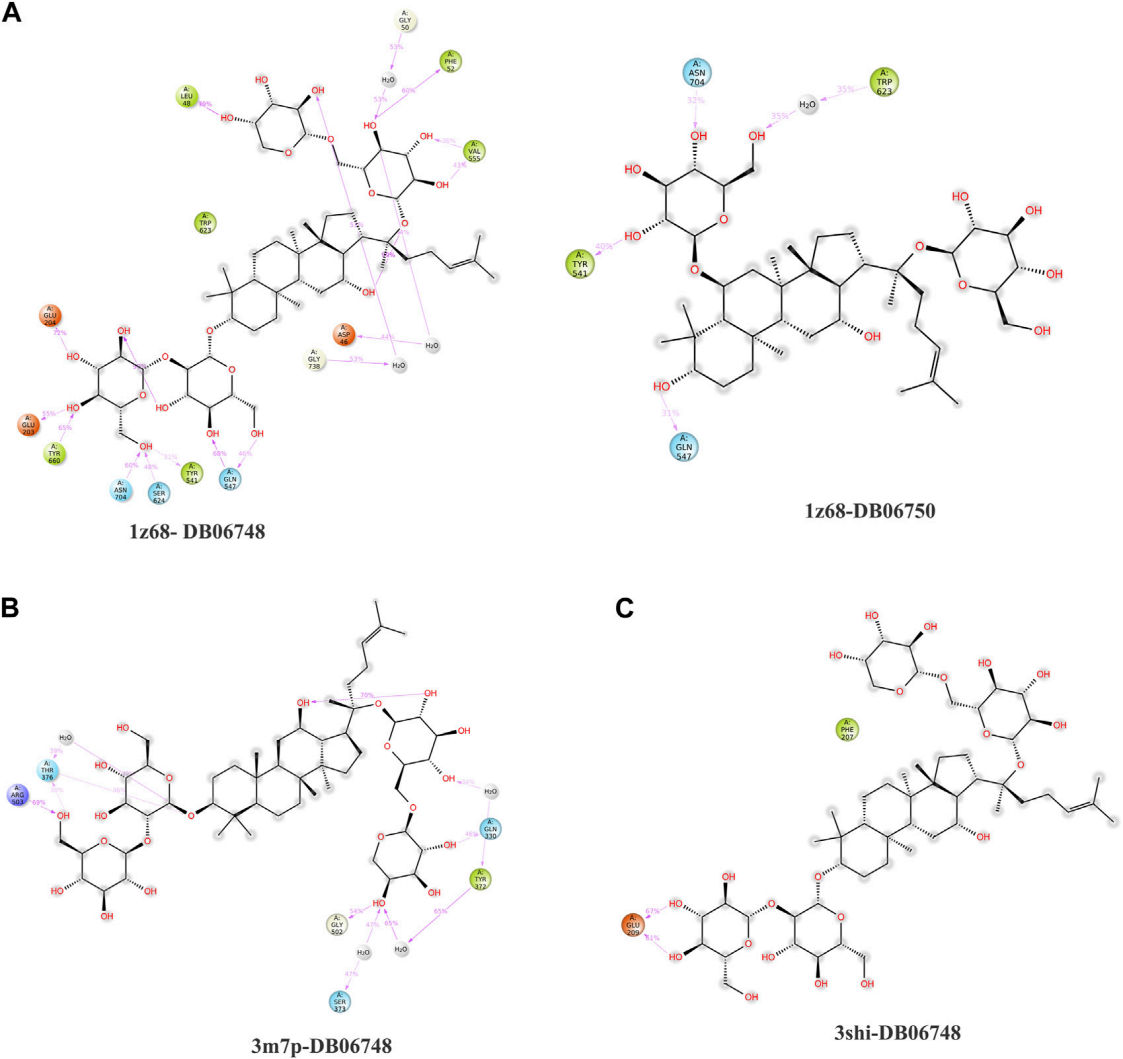
**(A–C)**: A schematic of detailed ligand atom interactions with the protein residues. Interactions that occur more than 30.0% of the simulation time in the selected trajectory (0.00 through 100.00 nsec).

The ligand-receptor complex’s stability can be forecast by RMSD readings. A minor adjustment to the RMSD could result in increased binding stability. Figures 8A–C displays the RMSD of the four complexes. Figures 9A–C depict the representation of interactions as H-bonds, hydrophobic, ionic, and water bridges. The number of distinct interactions the protein has with the ligand overall during the trajectory is displayed in the top panel. The residues that interact with the ligand in each trajectory frame are shown in the bottom panel. According to the scale to the right of the plot, some residues have multiple specific contacts with the ligand, which is depicted by a darker orange color. These interactions are shown in Figures 10A–C. Moreover, the schematic detail of the ligand-atom interaction with protein residues are shown in Figures 11A–C.

## 4 Discussion

Cancer comes from gene dysfunction, leading to cell proliferation mutations and irregularly affecting cell growth. Different oncogenes families can affect cancer proliferation as RAS, especially in human genes. The specific role of the RAS family is unclear, but by further studying oncogenes, it becomes more likely that the progression of cancer could be stopped (Benisty, Weber, Hernandez-Alias, Schaefer and Serrano, 2020). Oral cancer is one of the most perilous types because of the alteration of genes related to some behaviors. Many studies state that the main reason for gene alteration and silent mutation in oral cancer is having bad habits such as smoking and chewing tobacco. One of the reasons for the difficulty of studying the genetics of oral cancer is its type, being related to solid tumors that are not stable (Ali et al., 2017). As there are no specific biomarkers for oral cancer, this *in silico* study could help in the prognosis and diagnosis of the disease.

Molecular docking studies could lead to drug discovery. MD simulation has given many possibilities for novel drugs and drug repurposing to be used in defense of cancer (Yalcin-Ozkat, 2021). As it is widely used in Chinese medicines, Lin et al. examined hydroxychloroquine (HQ) to know more about its mechanism against prostate cancer using molecular docking (Lin et al., 2022).

Our study’s molecular docking results show high docking scores between the three target proteins (FAP, FN1, and MMP1) and ginsenoside C & Rg1. This creates four stable complexes (1z68- DB06748, 1z68-DB06750, 3m7p-DB06748, and 3shi-DB06748). The three (FAP, FN1, and MMP1) overexpressed genes show a significant potentiality for oral cancer development (Wang et al., 2014; Yang et al., 2021; Wu et al., 2022). The suggested way to control oral cancer is to inhibit and downregulate the targeted genes.

Using the DrugBank database as a ligand library, the approved drugs were repurposed to be used against oral cancer targets. The results show the interaction with ginsenoside derivatives, a natural base product that showed an effect against certain cancer types. The main role of ginsenosides is stopping the cell cycle by inducing apoptosis and inhibiting angiogenesis. Ginsenoside has attracted considerable attention for its use in cancer treatments (Wang, Anderson, Du, He and Yuan, 2016). Tang et al. showed that ginsenoside Rg1 plays a vital role in cell proliferation inhibition in acute myeloid leukemia (LAM) patients (Tang et al., 2020). Moreover, ginsenoside Rg1 significantly influences breast cancer treatment by inhibiting the expressed genes (Li et al., 2014).

The docking results show that the interaction between the molecules and the particular amino acids might inhibit the expression of our targeted proteins (FAP, FN1, and MMP1). The MD simulation process gave an accurate image of the possibility of oral cancer target inhibition. The protein-ligand complexes show stability under the physiological environment, creating significant possibilities in oral cancer treatment. This study not only refers to oral cancer treatment but also provides a comprehensive overview of the prospective targets.

## 5 Conclusion

In this study, we focused on oral cancer, which threatens future generations and is increasing rapidly because of a lack of information and many other factors, such as drinking alcohol, smoking, chewing tobacco, viruses, and other habits and infections. A person’s lifestyle may play an important role in the development of oral cancer symptoms. However, new factors have been identified in the attempt to avoid oral cancer, and new information and the latest treatment methods could save more lives. Molecular docking and simulation could help minimize time and cost in this battle. We chose three overexpressed genes (FAP, FN1, and MMP1) that could have potential regarding their presence in oral cancer patients. The *in silico* inhibition of these proteins could help develop treatments. This study aimed to find a repurpose for herbal medicine such as ginsenoside C and Rg1 in oral cancer treatment. The results show good interaction and physiological stability, which could be used for further experimental research in treatments for oral cancer.

## Data Availability

Publicly available datasets were analyzed in this study. This data can be found here: https://go.drugbank.com/ https://www.rcsb.org/.

## References

[B1] Abati S. Bramati C. Bondi S. Lissoni A. Trimarchi M. (2020). Oral cancer and precancer: A narrative review on the relevance of early diagnosis. Int. J. Environ. Res. Public Health 17 (24), 9160. 10.3390/ijerph17249160 33302498 PMC7764090

[B2] Ali J. Sabiha B. Jan H. U. Haider S. A. Khan A. A. Ali S. S. J. O. (2017). Genetic etiology of oral cancer. Oral Oncol. 70, 23–28. 10.1016/j.oraloncology.2017.05.004 28622887

[B3] Benisty H. Weber M. Hernandez-Alias X. Schaefer M. H. Serrano L. (2020). Mutation bias within oncogene families is related to proliferation-specific codon usage. Proc. Natl. Acad. Sci. U. S. A. 117 (48), 30848–30856. 10.1073/pnas.2016119117 33199641 PMC7720162

[B4] Berman H. M. Westbrook J. Feng Z. Gilliland G. Bhat T. N. Weissig H. (2000). The protein Data Bank. Nucleic Acids Res. 28 (1), 235–242. 10.1093/nar/28.1.235 10592235 PMC102472

[B5] Chow E. Rendleman C. A. Bowers K. J. Dror R. O. Hughes D. H. Gullingsrud J. (2008). Desmond performance on a cluster of multicore processors. New York, NY, USA: Columbia University.

[B35] D'Souza S. Addepalli V. (2018). Preventive measures in oral cancer: an overview. Biomed. Pharmacother. 107, 72–80. 10.1016/j.biopha.2018.07.114 30081204

[B6] Deng J. Yang Z. Ojima I. Samaras D. Wang F. (2022). Artificial intelligence in drug discovery: applications and techniques. Brief. Bioinform 23 (1), bbab430. 10.1093/bib/bbab430 34734228

[B7] Ferreira L. G. Dos Santos R. N. Oliva G. Andricopulo A. D. (2015). Molecular docking and structure-based drug design strategies. Molecules 20 (7), 13384–13421. 10.3390/molecules200713384 26205061 PMC6332083

[B56] Ghantous Y. Abu Elnaaj I. (2017). Global incidence and risk factors of oral cancer. Harefuah 156 (10), 645–649. 29072384

[B57] Gholizadeh P. Eslami H. Yousefi M. Asgharzadeh M. Aghazadeh M. Kafil H. S. (2016). Role of oral microbiome on oral cancers, a review. Biomed. Pharmacother. 84, 552–558. 10.1016/j.biopha.2016.09.082 27693964

[B8] Li L. Wang Y. Qi B. Yuan D. Dong S. Guo D. (2014). Suppression of PMA-induced tumor cell invasion and migration by ginsenoside Rg1 via the inhibition of NF-κB-dependent MMP-9 expression. Oncol. Rep. 32 (5), 1779–1786. 10.3892/or.2014.3422 25174454 PMC4203332

[B9] Li Z. Su C. Ding B. J. E. R. M. P. S. (2019). Molecular dynamics simulation of β-adrenoceptors and their coupled G proteins. Eur. Rev. Med. Pharmacol. Sci. 23 (14), 6346–6351. 10.26355/eurrev_201907_18458 31364142

[B10] Lin X. Li X. Lin X. (2020). A review on applications of computational methods in drug screening and design. Molecules 25 (6), 1375. 10.3390/molecules25061375 32197324 PMC7144386

[B11] Lin Z. Zhang Z. Ye X. Zhu M. Li Z. Chen Y. (2022). Based on network pharmacology and molecular docking to predict the mechanism of Huangqi in the treatment of castration-resistant prostate cancer. PLOS ONE 17 (5), e0263291. 10.1371/journal.pone.0263291 35594510 PMC9122509

[B12] Lopez-Lopez J. Omana-Cepeda C. Jane-Salas E. (2015). Oral precancer and cancer. Med. Clin. Barc. 145 (9), 404–408. 10.1016/j.medcli.2014.11.014 25638423

[B58] Macfarlane G. J. Boyle P. Evstifeeva T. V. Robertson C. Scully C. (1994). Rising trends of oral cancer mortality among males worldwide: the return of an old public health problem. Cancer Causes Control 5, 259–265. 10.1007/BF01830246 8061175

[B13] Napier S. S. Speight P. M. (2008). Natural history of potentially malignant oral lesions and conditions: an overview of the literature. J. Oral Pathol. Med. 37 (1), 1–10. 10.1111/j.1600-0714.2007.00579.x 18154571

[B14] Nariai Y. Mishima K. Yoshimura Y. Sekine J. (2011). FAP-1 and NF-κB expressions in oral squamous cell carcinoma as potential markers for chemo-radio sensitivity and prognosis. Int. J. Oral Maxillofac. Surg. 40 (4), 419–426. 10.1016/j.ijom.2010.10.020 21176871

[B15] Peng Y. Yin D. Li X. Wang K. Li W. Huang Y. (2023). Integration of transcriptomics and metabolomics reveals a novel gene signature guided by FN1 associated with immune response in oral squamous cell carcinoma tumorigenesis. J. Cancer Res. Clin. Oncol. 149, 6097–6113. 10.1007/s00432-023-04572-x 36656379 PMC11797619

[B16] Pinzi L. Rastelli G. (2019). Molecular docking: shifting paradigms in drug discovery. Int. J. Mol. Sci. 20 (18), 4331. 10.3390/ijms20184331 31487867 PMC6769923

[B17] Rufer A. C. (2021). Drug discovery for enzymes. Drug Discov. Today 26 (4), 875–886. 10.1016/j.drudis.2021.01.006 33454380

[B18] Saikia S. Bordoloi M. (2019). Molecular docking: challenges, advances and its use in drug discovery perspective. Curr. Drug Targets 20 (5), 501–521. 10.2174/1389450119666181022153016 30360733

[B59] Sciubba J. J. (2001). Oral cancer: the importance of early diagnosis and treatment. Am. J. Clin. Dermatol 2, 239–251. 10.2165/00128071-200102040-00005 11705251

[B19] Smith D. A. Di L. Kerns E. H. (2010). The effect of plasma protein binding on *in vivo* efficacy: misconceptions in drug discovery. Nat. Rev. Drug Discov. 9 (12), 929–939. 10.1038/nrd3287 21119731

[B20] Srivastava N. Garg P. Srivastava P. Seth P. K. (2021). A molecular dynamics simulation study of the ACE2 receptor with screened natural inhibitors to identify novel drug candidate against COVID-19. PeerJ 9, e11171. 10.7717/peerj.11171 33981493 PMC8074842

[B21] Syed M. Flechsig P. Liermann J. Windisch P. Staudinger F. Akbaba S. (2020). Fibroblast activation protein inhibitor (FAPI) PET for diagnostics and advanced targeted radiotherapy in head and neck cancers. Eur. J. Nucl. Med. Mol. Imaging 47 (12), 2836–2845. 10.1007/s00259-020-04859-y 32447444 PMC7567680

[B22] Tang Y. L. Zhang C. G. Liu H. Zhou Y. Wang Y. P. Li Y. (2020). Ginsenoside Rg1 inhibits cell proliferation and induces markers of cell senescence in CD34+CD38- leukemia stem cells derived from KG1α acute myeloid leukemia cells by activating the sirtuin 1 (SIRT1)/Tuberous sclerosis complex 2 (TSC2) signaling pathway. Med. Sci. Monit. 26, e918207. 10.12659/MSM.918207 32037392 PMC7032532

[B60] Tarapan S. Matangkasombut O. Trachootham D. Sattabanasuk V. Talungchit S. Paemuang W. (2019). Oral Candida colonization in xerostomic postradiotherapy head and neck cancer patients. Oral Dis. 25 (7), 1798–1808. 10.1111/odi.13151 31257663

[B23] Togre N. S. Vargas A. M. Bhargavi G. Mallakuntla M. K. Tiwari S. (2022). Fragment-based drug discovery against mycobacteria: the success and challenges. Int. J. Mol. Sci. 23 (18), 10669. 10.3390/ijms231810669 36142582 PMC9500838

[B24] van der Waal I. (2010). Potentially malignant disorders of the oral and oropharyngeal mucosa; present concepts of management. Oral Oncol. 46 (6), 423–425. 10.1016/j.oraloncology.2010.02.016 20308005

[B25] Wang C. Z. Anderson S. Du W. He T. C. Yuan C. S. (2016). Red ginseng and cancer treatment. Chin. J. Nat. Med. 14 (1), 7–16. 10.3724/SP.J.1009.2016.00007 26850342

[B26] Wang H. Wu Q. Liu Z. Luo X. Fan Y. Liu Y. (2014). Downregulation of FAP suppresses cell proliferation and metastasis through PTEN/PI3K/AKT and Ras-ERK signaling in oral squamous cell carcinoma. Cell Death Dis. 5 (4), e1155. 10.1038/cddis.2014.122 24722280 PMC5424105

[B27] Wishart D. S. Knox C. Guo A. C. Shrivastava S. Hassanali M. Stothard P. (2006). DrugBank: A comprehensive resource for *in silico* drug discovery and exploration. Nucleic Acids Res. 34, D668–D672. 10.1093/nar/gkj067 16381955 PMC1347430

[B28] Wooller S. K. Benstead-Hume G. Chen X. Ali Y. Pearl F. M. G. (2017). Bioinformatics in translational drug discovery. Biosci. Rep. 37 (4). 10.1042/BSR20160180 PMC6448364 28487472

[B29] Wu T. Jiao Z. Li Y. Su X. Yao F. Peng J. (2022). HPRT1 promotes chemoresistance in oral squamous cell carcinoma via activating MMP1/PI3K/akt signaling pathway. Cancers (Basel) 14 (4), 855. 10.3390/cancers14040855 35205603 PMC8870334

[B30] Yalcin-Ozkat G. (2021). Molecular modeling strategies of cancer multidrug resistance. Drug Resist Updat 59, 100789. 10.1016/j.drup.2021.100789 34973929

[B31] Yang W. Zhou W. Zhao X. Wang X. Duan L. Li Y. (2021). Prognostic biomarkers and therapeutic targets in oral squamous cell carcinoma: A study based on cross-database analysis. Hereditas 158 (1), 15. 10.1186/s41065-021-00181-1 33892811 PMC8066950

[B32] Ying Y. Liu D. Zhao Y. Zhong Y. Xu X. Luo J. (2022). LINC01116 promotes migration and invasion of oral squamous cell carcinoma by acting as a competed endogenous RNA in regulation of MMP1 expression. Comput. Math. Methods Med. 2022, 2857022. 10.1155/2022/2857022 35756415 PMC9232319

[B33] Zhang W. Huang X. Huang R. Zhu H. Ye P. Lin X. (2022). MMP1 overexpression promotes cancer progression and associates with poor outcome in head and neck carcinoma. Comput. Math. Methods Med. 2022, 3058342. 10.1155/2022/3058342 36105241 PMC9467809

[B34] Zhou W. H. Du W. D. Li Y. F. Al-Aroomi M. A. Yan C. Wang Y. (2022). The overexpression of Fibronectin 1 promotes cancer progression and associated with M2 macrophages polarization in head and neck squamous cell carcinoma patients. Int. J. Gen. Med. 15, 5027–5042. 10.2147/IJGM.S364708 35607361 PMC9123938

