# Supplementary material for: Using CADD tools to inhibit the overexpressed genes FAP, FN1, and MMP1 by repurposing ginsenoside C and Rg1 as a treatment for oral cancer
Source: Front Mol Biosci. 2023 Oct 23;10:1248885. doi: 10.3389/fmolb.2023.1248885 (PMC10627001; doi:10.3389/fmolb.2023.1248885)
Supplement: Supplementary file 1 [file Table1.DOCX]

| **No.** | **Drugbank ID** | **Macro-molecule** | **Docking score** | **Bond types of interaction with amino acids** | | | | | |
| --- | --- | --- | --- | --- | --- | --- | --- | --- | --- |
|  |  |  |  | **H-bond** | **Halogen bond** | **Metal coordination** | **Pi-Pi stacking** | **Salt bridge** | **Pi-cation** |
| **1** | DB06748 | FAP | -12.142 | LEU 48, ILE 47, TYR 541, VAL 555, GLU 203, GLH 204, TYR 656 |  |  |  |  |  |
| **2** | DB06750 |  | -11.303 | GLY 735, TYR 745, TYR 656, GLU 203, ARG 550 |  |  |  |  |  |
| **3** | DB06748 | FN1 | -14.201 | ALA 465, TYR 372, SER 373, ARG 369, THR 375, GLU 505, GLY 502 |  |  |  |  |  |
| **4** | DB06748 | MMP1 | -9.415 | LYS (A:136), GLY (A;244), ASP (A:129), GLN (A:247), TYR (G:121) |  |  |  |  |  |

**Supplementary Table 1**: The DrugBank database top ligands interaction with FAP, FN1, and MMP1, showing the interacted amino acid
